# Pilot-scale spiral wound membrane assessment for THM precursor rejection from upland waters

**DOI:** 10.1080/01496395.2016.1162807

**Published:** 2016-03-23

**Authors:** D. Golea, S. Sutherland, P. Jarvis, S. J. Judd

**Affiliations:** ^a^ Scottish Water, Edinburgh, Scotland; ^b^ Cranfield Water Science Institute, Cranfield University, Bedfordshire, UK; ^c^ Gas Processing Center, Qatar University, Doha, Qatar

**Keywords:** Nanofiltration, natural organic matter, potable water, triahlomethanes, ultrafiltration

## Abstract

The outcomes of a pilot-scale study of the rejection of trihalomethanes (THMs) precursors by commercial ultrafiltration/nanofiltration (UF/NF) spiral-wound membrane elements are presented based on a single surface water source in Scotland. The study revealed the expected trend of increased flux and permeability with increasing pore size for the UF membranes; the NF membranes provided similar fluxes despite the lower nominal pore size. The dissolved organic carbon (DOC) passage decreased with decreasing molecular weight cut-off, with a less than one-third the passage recorded for the NF membranes than for the UF ones.

The yield (weight % total THMs per DOC) varied between 2.5% and 8% across all membranes tested, in reasonable agreement with the literature, with the aromatic polyamide membrane providing both the lowest yield and lowest DOC passage. The proportion of the hydrophobic (HPO) fraction removed was found to increase with decreasing membrane selectivity (increasing pore size), and THM generation correlated closely (*R* ^2^ = 0.98) with the permeate HPO fractional concentration.

## Introduction

It has been recognised for more than 40 years that the reaction of natural organic matter (NOM) with chlorine generates chlorinated disinfection by-products (DPBs) generally and trihalomethanes (THMs) specifically.^[^ ^1^ ^]^ In the UK the prescribed concentration value of THMs in potable water is currently 100 µg L^−1.[^ ^2^ ^]^ An established option for addressing this issue is the removal of the NOM using membranes. A large amount of research has been conducted to ascertain the precise characteristics and chemical functional groups within the NOM dissolved organic carbon (DOC) responsible for THM generation, both upstream and downstream of a membrane separation process.^[^ ^3^ ^–^ ^5^ ^]^ A review of the recent available literature suggests that the yield of total THMs (tTHMs) in treated water tends to be in the range of 2–8% THMs per DOC.^[^ ^6^ ^]^ Thus, whilst the residual DOC level provides an indication of THM formation propensity (THMFP in µg L^−1^), such that DOC passage through the membrane is a useful performance indicator, the yield is subject to significant variation.

**Figure 1.**
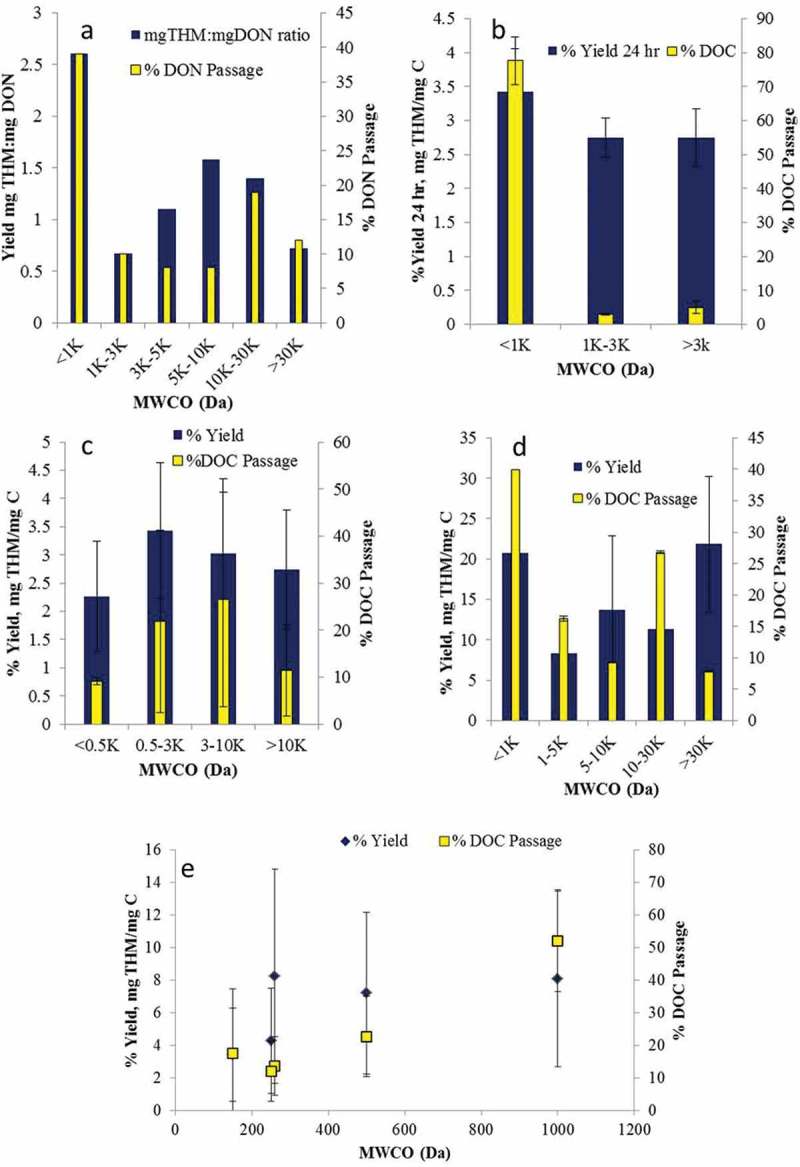
% Yield and % DOC passage determined from reported literature data: a [10], b [8], c [4], d [9], e [11].

Membranes of appropriate selectivity—generally in the tight ultrafiltration (UF)/loose nanofiltration (NF) region—have been shown to be reasonably effective for removing DOC, and thus THM precursors.^[^ ^6^ ^,^ ^7^ ^]^ However, an examination of available data for NF/UF membranes (Table 1) reveals widely varying trends in DOC passage and yield with membrane selectivity. In many cases^[^ ^4^ ^,^ ^8^ ^,^ ^9^ ^]^ there is no recognisable trend in either DOC or yield with perm-selectivity as represented by the molecular weight cut-off, or MWCO (Fig. 1b–1d). Data are very highly scattered, with relative standard deviation (SD) values of between 5% and 41% and 17–67%, for DOC passage and yield respectively—as reflected in the error bars in Fig. 1d. Dissolved organic nitrogen (DON) passage similarly does not correlate with MWCO (Fig. 1a ^[^ ^10^ ^]^). It is only in one case (Fig. 1e ^[^ ^11^ ^]^) that there appears to be the expected relationship of increasing DOC passage with increasing membrane MWCO. This report, based on 10 different feed waters predominantly from the Murcia region of Spain, also suggests an increase in yield with increasing MWCO, albeit with anomalously high yield values for one particular membrane (at 260 Da MWCO). Against this, three of the other papers^[^ ^4^ ^,^ ^8^ ^,^ ^9^ ^]^ indicate that the highest yield is obtained at the lowest MWCO (Table 1). Outcomes are significantly affected by the acknowledged seasonal variations in NOM characteristics.^[^ ^12^ ^]^

**Table 1.** Literature data, yield and DOC passage.

Water source(s)	Membrane(s)	%DOC passage vs. MWCO		%DOC passage vs. yield		Refs.
		Gradient	R^2^	Gradient	R^2^	
Crevillente	*Millipore YM1*	0.036	0.85	−0.015	0.0001	[11]
Guadalest	*Millipore YC05*	0.058	0.97	0.38	0.37	
La Pedera	*DOW NF270*	0.049	0.94	0.0044	0.0002	
Tibi	*DOW NF90*	0.066	0.80	0.45	0.094	
Villajoyosa	*Alfa Laval NFT50*	0.013	0.04	0.079	0.048	
Mayayo		0.04	0.96	0.91	0.79	
Regueron		0.067	0.98	0.79	0.54	
Reina		0.036	0.87	0.49	0.058	
Segura River		0.038	0.98	0.38	0.49	
Taibilla		0.059	0.85	−0.053	0.025	
Yellow River	*Millipore*	−6.01	0.58	−0.032	0.046	[9]
Danjiangkou Reservoir	*Millipore YM10*	−4.7	0.25	0.2	0.44	
Repentigny	*Millipore YM10*	−2.4	0.58	−0.48	0.97	[4]
Waco	*Millipore YM3*	2.3	0.04	1.9	0.85	
Winnipeg	*Millipore YC 05*	3.67	0.11	1.16	0.057	
Terkos raw	*Millipore YM*	−31	0.7	5.4	0.97*	[8]
- post ozonation		−35	0.73	8.5	0.91*	
- post coag/flocc		−41	0.74	30	0.90*	
- post filtration		−38	0.74	6.2	0.44*	

*24 h THM FP method.

THMFP and yield studies in this area have often encompassed chemical fractionation.^[^ ^13^ ^–^ ^17^ ^]^ It is generally considered that the hydrophobic (HPO) fraction of the NOM, associated with humic acids, generates higher yields than the hydrophilic (HPI) fraction,^[^ ^4^ ^,^ ^9^ ^,^ ^16^ ^]^ though exceptions to this observation have been reported.^[^ ^18^ ^]^ Whilst it is well known that the HPO fraction is preferentially removed by conventional clarification, the remaining HPI fraction is nonetheless capable of generating THMs.^[^ ^4^ ^,^ ^18^ ^]^ There is no evidence of significant differences in yield from HPI and transphilic (TPI) fractions.^[^ ^4^ ^]^

Available reported data indicate unpredictable and sometimes contradictory trends in DOC passage and yield. DOC molecular size appears to be a poor indicator of yield across different waters, and trends are only likely to be discernible for single water sources. The HPO fraction is generally recognised as representing the most reactive component of the NOM generating THMs,^[^ ^4^ ^,^ ^9^ ^,^ ^16^ ^]^ but there is again little consistency across different studies regarding the actual yield. The aim of this work is to assess (a) the DOC removal capability of membranes of different MWCO and/or selectivity ratings, and (b) trends in THM yield with membrane characteristics, based on a single feedwater. It is of further interest to establish whether the classical hydrophobicity/hydrophilicity chemical fractionation of the organic matter is of significance regarding yield.

## Materials and methods

### Materials

**Figure 2.**
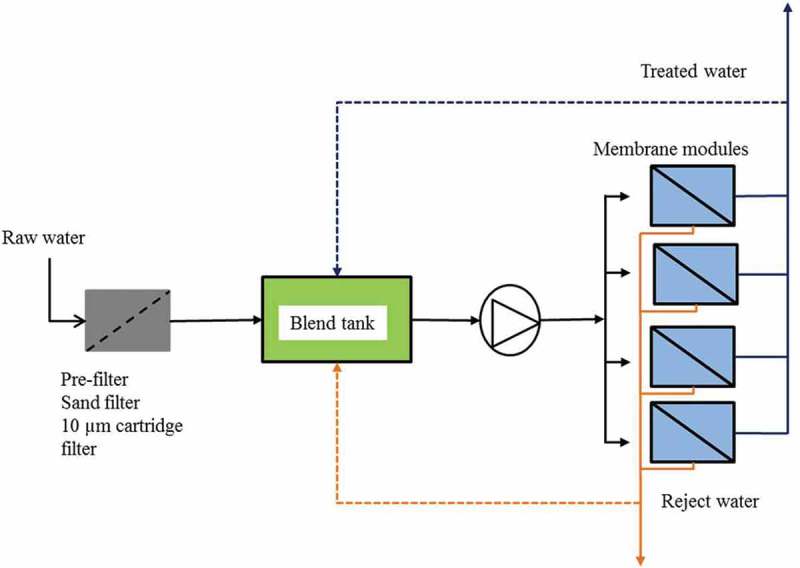
Pilot test rig configuration.

The pilot plant (Fig. 2) was based at a water treatment works in the Scottish Highlands, fed with surface water of low dissolved solids and relatively high DOC (Table 2). It comprised four streams, each fitted with a standard commercial 1 m long, 100 mm diameter spiral-wound membrane element housed in a glass-reinforced plastic (GRP) pressure vessel. A single pump (30 m^3^ h^−1^ flow, 5.9–6.2 bar feed pressure depending on temperature) was used to feed all four streams, with flows to each stream metered by individual control valves, and the conversion set at ~10% per stream. The feed water was fed to a blend tank where it was mixed with the retentate stream from the four individual streams, providing a means to increase the feed organics concentration by a factor of ~3 (Table 2) to replicate the maximum challenge to the membranes when operating at a total conversion of 80% at full scale. On reaching the target concentration factor of 3 the plant was operated as a closed loop, i.e. recirculating both feed and permeate, so as to sustain the target feed organic carbon concentration, for a period of 48 hours. Two 9-day campaigns were conducted.

**Table 2.** Feed and blend water characteristics.

Parameter	Campaign 1				Campaign 2			
	Raw		Blend		Raw		Blend	
	Mean	SD	Mean	SD	Mean	SD	Mean	SD
*Colour (mg L^−1^ Pt/Co)*	43	19	102	35	57	8	144	25
*DOC (mg L^−1^)*	4.2	0.5	17	5.1	7.4	1.8	18	3.2
*UV254 (m^−1^)*	27	10	68	20	42	12	88	14
*SUVA (L mg^−1^m^−1^)*	6.5	2.9	3.8	0.77	5.9	1.9	5	0.11
*Conductivity (mS cm^−1^,20°C)*	29	1.2	62	10	24	1.8	40	6.2
*pH*	6.4	0.07	7.5	0.07	6.3	0.1	6.8	0.2
*Calcium (mgCa L^−1^)*	1.5	0.1	4.8	1.2	1.2	0.05	2.8	0.5
*Manganese (µgMn L^−1^)*	23	16	38	6	16	0.7	31	4.4
*Iron (µgFe L^−1^)*	350	7.9	53	9	370	12	250	25
*Temperature (°C)*	9.7	1.3	23	12	9.2	0.7	21	14
*Turbidity (NTU)*	0.6	0.23	0.2	0	0.58	0.3	0.27	0.06

The specifications of the membrane material investigated varied from a UF of 8000 Da MWCO to an NF of 260 Da (Table 3). Membranes #1 and #6 were used in both campaigns as controls (Table 3), and the remaining membranes as test products. Membranes classified as NF were assumed to have an element of charge rejection, as opposed to purely physical rejection based on molecular size for the UF membranes. Membrane materials included cellulose acetate and sulphonated polyethersulphone, these being less widely studied than the classical polyamide/polypiperazine NF membranes.

**Table 3.** Spiral wound membrane MWCO and material characteristics.

Membrane	Campaign	Membrane MWCO (Da)	Average % salt rejection	Membrane material	Membrane type
*ID*					
*#1*	1st,2nd	260	97^a^	Polypiperazine thin-film (PPA)	NF
*#2*	1st	700	85^b^	Cellulose acetate (CA)	NF
*#3*	1st	1000	50^b^	Sulphonated polyethersulphone (S-PS)	UF
*#4*	2nd	2000	N/A	Cellulose acetate (CA)	UF
*#5*	2nd	3000	20^b^	Sulphonated polyethersulphone (S-PS)	UF
*#6*	1st, 2nd	8000	N/A	Cellulose acetate (CA)	UF

Salt rejection measure using ^a^MgSO_4_ and ^b^NaCl, respectively; MWCO determined using dextran; N/A – not available.

### Methods

#### Pilot plant operation

Two campaigns, each of nine days, were undertaken. Samples of raw, filtered, blend and permeate water were collected at 48-hour intervals and delivered on the same day to the Scottish Water laboratories. Analysis was undertaken for colour, turbidity, UV_254_ transmittance, DOC and NOM fractionation by adsorption, the latter based on a method adapted from Bessiere et al. (2009)^[^ ^19^ ^]^ and applied to single samples. The seven-day THM yield was determined by chlorinating all samples and using a modified form of the USEPA Method 551.1.^[^ ^20^ ^]^ All laboratory analyses followed methods routinely employed by Scottish Water Laboratories.^[^ ^21^ ^]^ Laboratory analyses were supplemented by on-site tests for colour (mg L^−1^ Pt) using a Hach Lange DR 3900 (UV_410_ absorption set programme measured in a 4-cm cuvette), turbidity (NTU) using a Hach 2100P portable turbidimeter, and temperature (°C) and pH using a Hach HQ30d flexy portable pH and temperature meter.

The recirculation of the retentate and permeate in a closed loop caused an increase in the feed water temperature increased to up to 34°C due to frictional forces. Membrane flux and permeability were normalised to 20°C using the viscosity correction equation:

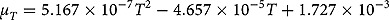

where 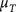 in kg m^−1^ s^−1^ is the viscosity at temperature 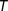 and 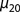 the value at 20°C (1.009 kg m^−1^ s^−1^). The normalised flux for 20°C (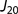) for a measured flux of 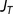 at temperature *T* is then given by

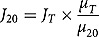

The normalised permeability (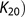 was determined from the normalised flux and recorded transmembrane pressure (TMP) in bar:

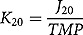

## Results and discussion

### Reproducibility

The assigned flux and measured permeability of the two control membranes were similar, despite the differences in selectivity, but the flux and permeability values for the second campaign were somewhat lower than the first. For the first campaign the flux values of the two controls (Membranes #1 and #6) were 57 ± 7 and 57 ± 3 LMH, respectively, compared with 46 ± 9 and 49 ± 3 LMH for the second campaign. The corresponding permeability values were 10 ± 1.6 and 10 ± 0.8 LMH/bar for Campaign 1 and 7.8 ± 2.1 and 8.2 ± 1.0 LMH/bar for Campaign 2. The lower values recorded for the second campaign presumably reflected the more highly fouling nature of the feedwater. Fouling is normally associated with the colour, UV_254_ and/or turbidity levels—all of which were 29–41% higher on average in the blend water for Campaign 2 than that for Campaign 1.

Reproducibility of the %yield across the two campaigns was good, with the Membrane #1 values being 2.5 ± 2% and 2.4 ± 0.3% for the first and second campaigns and the corresponding Membrane #6 values being 7.0 ± 2% and 8.2 ± 2%. Conversely, the %DOC passage values were more unpredictable, with Membrane #1 values of 7.0 ± 4% and 3.9 ± 1% and Membrane #6 values of 24 ± 9% and 14 ± 2% for the two campaigns, respectively, giving an overall relative standard deviation of 40–41%. This trend of reduced DOC passage is consistent with that of the higher fouling propensity, reflected in the flux and permeability trends.

### Flux and permeability

**Figure 3.**
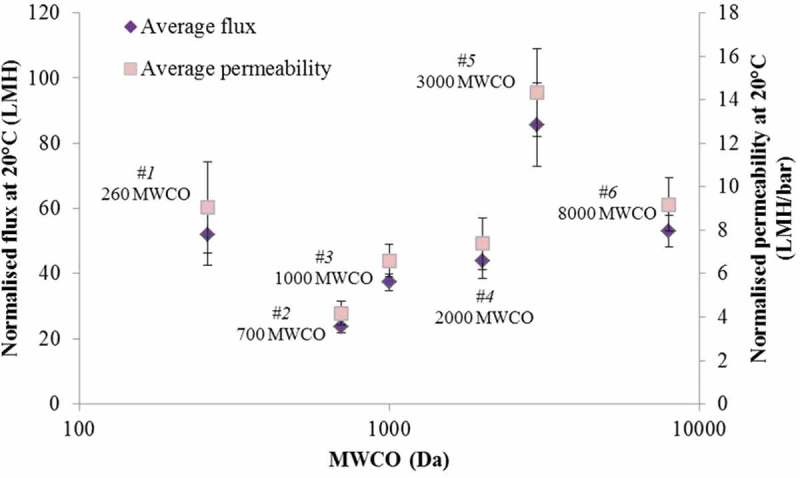
Normalised flux and permeability at 20°C for the six membrane products.

Fluxes varied widely according to the membrane selectivity (expressed as MWCO) and material characteristics (Fig. 3). The only polypiperazine membrane tested, and the most selective in terms of the stated MWCO (260 Da), provided a normalised flux (52 LMH) and permeability (9.1 LMH/bar) comparable with the least selective 8000 Da MWCO membrane. The significant decrease in selectivity between the 2000 and 8000 Da MWCO cellulose acetate (CA) membranes was not reflected in a commensurate increase in permeability (7.4 and 9.1 LMH/bar, respectively). The recorded permeabilities are higher than values of 3.3–4 LMH/bar previously reported for various pilot- and full-scale NF plants,^[^ ^8^ ^–^ ^10^ ^]^ probably due to the near-virgin state of the membranes used in the current study.

### Yield and DOC passage

**Figure 4.**
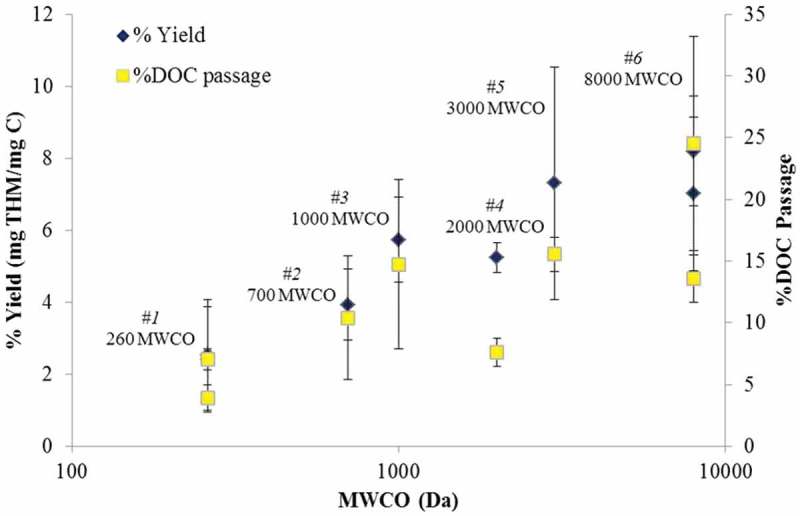
Yield and % DOC passage across the two campaigns, with two sets of data for the control membranes.

The %yield for the permeate samples across all the membranes tested ranged from 2.4 to 8.2, roughly according to selectivity expressed as MWCO (Fig. 4). Results were in good agreement with the range of yield previously recorded for Scottish surface waters across 35 full-scale membrane installations,^[^ ^6^ ^]^ despite the elevated temperatures of the current study. For the two NF membranes (denoted 260 and 700 Da MWCO) the permeate tTHM level was notably low, in accordance with the low recorded DOC passage (Fig. 6). The yield was found to increase with decreasing selectivity for both campaigns, corroborating previously reported trends.^[^ ^11^ ^]^ The correlation between yield and the specific UV_254_ absorbance (SUVA) was poor (*R* ^2^ = 0.69) (Table 4), supporting previous observations^[^ ^22^ ^–^ ^24^ ^]^ and suggesting that the permeate organic matter present is predominantly non-aromatic. Whereas UV_254_ and SUVA are often considered to be good THMFP indicators in waters having a DOC concentration and SUVA values above ~3 mg L^−1^ and ~4 L (mgC.m) ^−1^ respectively,^[^ ^16^ ^,^ ^20^ ^,^ ^26^ ^]^ for low SUVA values (<2 L (mgC.m)^−1^) the correlation is less valid. Measured SUVA values revealed the permeate from the most porous UF membranes (Membranes #5 and #6) to have the highest SUVA values (3.8–4.5 L (mgC.m) ^−1^) on average), compared with 1.4 or less on average for all other membranes (Table 4).

**Table 4.** Specific UV_254_ absorbance of measured permeate and blend water samples.

	Campaign 1									Campaign 2						
Sample	UV_254_	SD	DOC, (mg C) L^−1^	SD	SUVA, L (mg C m)^−1^	SD	Yield, µg THM (mg C)^−1^	SD	UV_254_	SD	DOC, (mg C) L^−1^	SD	SUVA, L (mg C m)^−1^	SD	Yield, µg THM (mg C)^−1^	SD
Raw	0.27	0.10	4.3	0.42	6.5	2.9	127	189	0.42	0.10	7.4	1.8	5.9	1.9	134	20
Filtered	0.25	0.11	4.0	0.20	6.2	2.5	109	23	0.40	0.13	7.2	1.8	5.7	1.9	128	14
Blend	0.68	0.20	18	4.63	3.8	0.77	112	19	0.89	0.14	18	3.2	5	0.11	155	26
*Membrane #1*	0.01	0.01	0.82	0.74	1.4	1.2	25	15	0.03	0.05	0.66	0.11	1	0.44	24	2.9
*Membrane #2*	0.01	0.00	1.6	0.80	0.83	0.45	39	9.9	–	–	–	–	–	–	–	–
*Membrane #3*	0.02	0.01	2.4	1.1	1	0.49	57	12	–	–	–	–	–	–	–	–
*Membrane #4*	–	–	–	–	–	–	–	–	0.02	0.01	1.3	0.23	1.7	0.5	52	4.2
*Membrane #5*	–	–	–	–	–	–	–	–	0.12	0.10	2.8	0.72	4.5	3.5	73	32
*Membrane #6*	0.07	0.02	4.2	1.4	3.8	0.59	70	21	0.09	0.09	2.4	0.43	3.8	3.2	82	15

**Figure 5.**
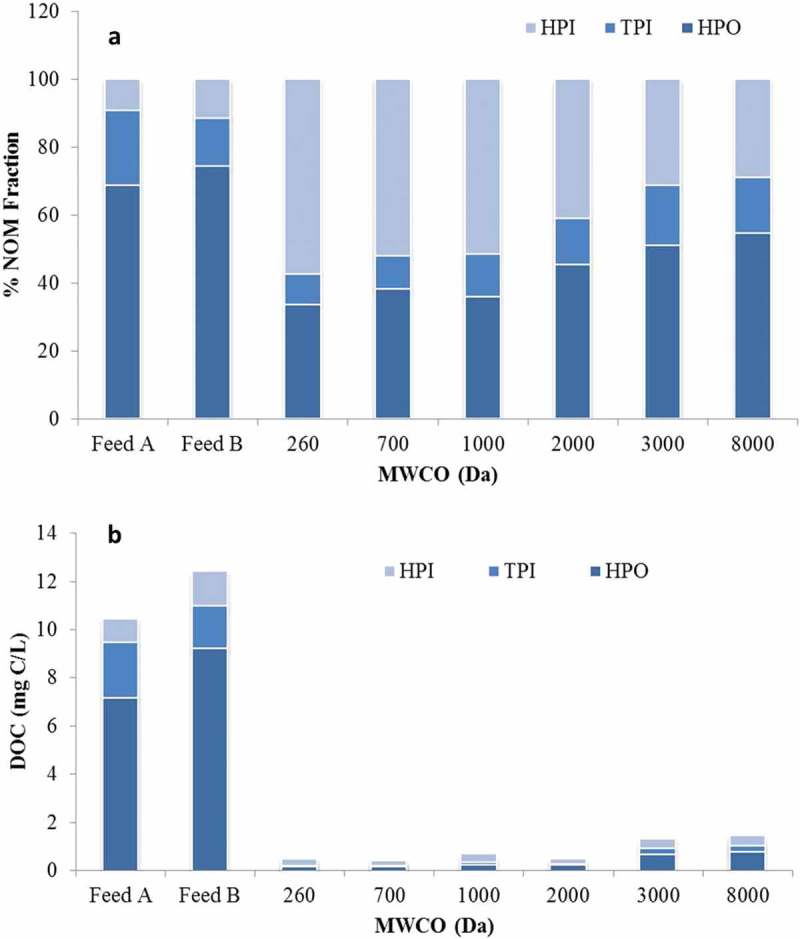
Organic fractions, membrane permeate (a) percentage, and (b) absolute concentration. Feed A refers to the 260, 700, 1000 and 8000 Da MWCO membranes, Feed B to 2000 and 3000 Da MWCO membranes.

**Figure 6.**
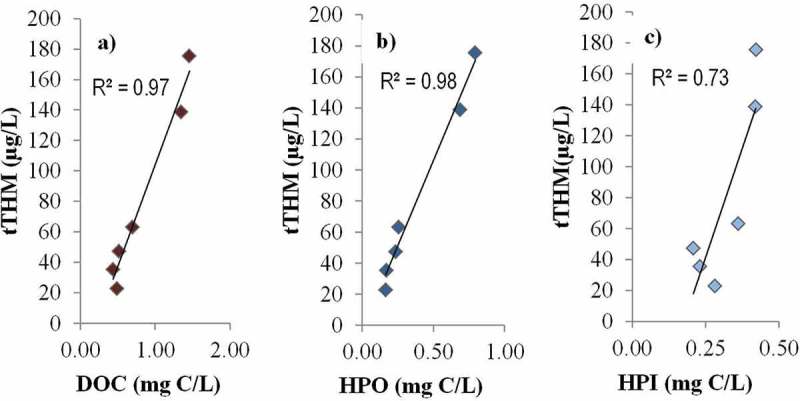
Total THMs vs. (a) recovered DOC, (b) hydrophobic (HPO) NOM fraction, and (c) hydrophilic (HPI) NOM fraction.

### NOM fractions

Measured concentrations of individual organic chemical fractions revealed the selectivity for the HPO fraction to increase with decreasing MWCO, with the NF membrane providing the greatest removal of this fraction (Fig. 5). As a result of this perm-selectivity the proportion of HPI organics permeating the membrane increased from 9% to 12% in the feed to a decreasing trend of between 29% and 57% in the permeate, the percentage increasing with increasing membrane selectivity. The yield of 2.4–2.5% THM/DOC for the most selective membrane, and thus the greatest permeate HPI proportion, is in good agreement with previously reported values^[^ ^4^ ^]^ based on Millipore membranes of 0.5, 3 and 10 kDa MWCO (Table 1). The HPI removal was highest for the most selective membranes, 260 and 700 Da for Membranes #1 and #2 respectively, providing 71% and 76% removal respectively, and lowest for the least selective 8000 Da MWCO membrane (57% removal). Similar removals of 71% of HPI acids have been reported for conventional clarification.^[^ ^27^ ^]^ The TPI NOM fraction is characterised by lower aromatic content than HPO^[^ ^28^ ^]^ and correspondingly lower SUVA values: it is comparable to HPI as a THM precursor in waters with low humic content.^[^ ^4^ ^]^

A good correlation (*R* ^2^ = 0.97–0.98) between the measured permeate tTHM and the DOC and the HPO fraction concentrations was observed across the six membranes tested (Fig. 6a,b). The corresponding correlation with the HPI fraction (Fig. 6c) was markedly weaker (*R* ^2^ = 0.73). Whilst these trends appear to corroborate those previously reported,^[^ ^29^ ^]^ the current data set is based on single rather than replicated measurements and relatively low carbon recovery (50–75%) by the extraction method. Notwithstanding this, there is apparently a closer correlation of tTHM with the HPO fraction than the HPI one.

## Conclusions

A pilot-scale study of the performance of candidate membranes for the removal of NOM for ameliorating THM formation in potable water treatment has revealed that:

- Flux and permeability trends vs. MWCO for the UF membranes followed the expected trend of decreasing flux with MWCO, the exception being the second most porous product Membrane #5. The NF membranes provided similar fluxes despite differences in the rated MWCO.
- Recorded flux values were markedly higher than those previously reported in the literature for full and pilot plants.
- DOC passage decreased with decreasing MWCO, an intuitive outcome which nonetheless conflicts with more unpredictable trends previously reported for NOM removal by membranes. The mean DOC passage of the two NF membranes tested was significantly lower—4–8% compared to 11–25%—than the UF membranes.
- The yield of THM also decreased with decreasing MWCO, varying between 2.5% and 8% in reasonable agreement with the range of values reported in the literature. Membrane #1—the only aromatic polyamide tested—provided both the lowest yield and the lowest DOC passage.
- The membrane selectivity for HPO increased with decreasing MWCO; the most highly selective NF membrane provided the largest proportional removal of the HPO fraction. The residual HPI fraction made up 29–57% of the total DOC, the proportion increasing with decreasing MWCO.
- The total THM concentration correlated well (*R* ^2^ > 0.97) with both the DOC and HPO fraction, with a much poorer correlation (*R* ^2^ = 0.73) with the HPI fraction.

## Funding

The financial and practical support of EPSRC (under the STREAM programme) and Scottish Water are gratefully acknowledged.
